# Early gut mycobiota and mother-offspring transfer

**DOI:** 10.1186/s40168-017-0319-x

**Published:** 2017-08-24

**Authors:** Kasper Schei, Ekaterina Avershina, Torbjørn Øien, Knut Rudi, Turid Follestad, Saideh Salamati, Rønnaug Astri Ødegård

**Affiliations:** 1 Department of Laboratory Medicine, Children’s and Women’s Health, Faculty of Medicine and Health Sciences, NTNU – Norwegian University of Science and Technology, Postboks 8905, 7491 Trondheim, Norway; 2 Department of Chemistry, Biotechnology and Food Science, NMBU – Norway University of Life Sciences, Ås, Norway; 3 Department of Public Health and Nursing, Faculty of Medicine and Health Science, NTNU – Norwegian University of Science and Technology, Trondheim, Norway; 4 ObeCe – Regional Centre for Obesity Research and Innovation, St. Olav’s University Hospital, Trondheim, Norway

**Keywords:** Gut microbiota, Mycobiota, Fungi, Newborn, Infant, Infant health, Probiotics

## Abstract

**Background:**

The fungi in the gastrointestinal tract, the *gut mycobiota*, are now recognised as a significant part of the gut microbiota, and they may be important to human health. In contrast to the adult gut mycobiota, the establishment of the early gut mycobiota has never been described, and there is little knowledge about the fungal transfer from mother to offspring.

**Methods:**

In a prospective cohort, we followed 298 pairs of healthy mothers and offspring from 36 weeks of gestation until 2 years of age (1516 samples) and explored the gut mycobiota in maternal and offspring samples. Half of the pregnant mothers were randomised into drinking probiotic milk during and after pregnancy. The probiotic bacteria included *Lactobacillus rhamnosus* GG (LGG), *Bifidobacterium animalis* subsp. *lactis* Bb-12 and *Lactobacillus acidophilus* La-5. We quantified the fungal abundance of all the samples using qPCR of the fungal internal transcribed spacer (ITS)1 segment, and we sequenced the 18S rRNA gene ITS1 region of 90 high-quantity samples using the MiSeq platform (Illumina).

**Results:**

The gut mycobiota was detected in most of the mothers and the majority of the offspring. The offspring showed increased odds of having detectable faecal fungal DNA if the mother had detectable fungal DNA as well (OR = 1.54, *p* = 0.04). The fungal alpha diversity in the offspring gut increased from its lowest at 10 days after birth, which was the earliest sampling point. The fungal diversity and fungal species showed a succession towards the maternal mycobiota as the child aged, with *Debaryomyces hansenii* being the most abundant species during breast-feeding and *Saccharomyces cerevisiae* as the most abundant after weaning. Probiotic consumption increased the gut mycobiota abundance in pregnant mothers (*p* = 0.01).

**Conclusion:**

This study provides the first insight into the early fungal establishment and the succession of fungal species in the gut mycobiota. The results support the idea that the fungal host phenotype is transferred from mother to offspring.

**Trial registration:**

Clinicaltrials.gov NCT00159523

**Electronic supplementary material:**

The online version of this article (doi:10.1186/s40168-017-0319-x) contains supplementary material, which is available to authorized users.

## Background

The fungi that populate the gastrointestinal tract (gut mycobiota) have recently been recognised as a substantial part of the gut microbiota and can be important for human health [1]. The adult gut mycobiota, which probably comprise approximately 13% of the gut microbial volume, consists of a species selection from approximately 140 different fungal genera [2, 3], with the most abundant ones being *Candida*, *Saccharomyces* and *Cladosporium* spp. [1].

Gut microbiota has been extensively studied over the last two decades. The Human Microbiome Project, or HMP [4], and the Metagenomics of the Human Intestinal Tract (MetaHIT) [5] have contributed greatly to our knowledge of the human microbial community structure, although no comprehensive and uniformly processed database can represent the human gut microbiome [6]. Fungal communities are far less studied. However, a positive association between the archaeon *Methanobrevibacter* and *Candida* with relative abundance differences in *Prevotella* might exist. Similarly, the relative abundance differences in *Bacteriodes* are associated with the archaeon *Nitrososphaera*, and they are negatively correlated with gut fungi [7].

The human gut mycobiota confers several physiological effects to the human body. These fungi consume nutrients and may facilitate nutrient extraction and assist in digestion through enzyme and vitamin production [7, 8]. The gut mycobiota is also essential as a form of antigen exposures to train the immune system and its responses. Through activating the fungus-specific pathogen-recognition receptors (PRRs) and adjacent mechanisms, defences against harmful pathogens and likewise a tolerance towards helpful commensals are formed [1, 9, 10].

However, for some humans, fungi can have unfavourable impacts, and the term *fungal dysbiosis* describes a state of unbalanced mycobiota associated with disease [11]. This phenomenon is most extensively studied in immunocompromised patients who regularly contract opportunistic commensal fungal infections [1] and in patients with obesity and inflammatory bowel disease (IBD) [12–15]. Obesity and metabolic disorders have been associated with the increased presence and abundance of *Saccharomycetes* spp., *Dipodascaceae* spp. and *Tremellomycetes* spp. [12]. Obesity and metabolic syndrome are pro-inflammatory states, and *Tremellomycetes* spp. are associated with higher inflammation levels. Accordingly, a lower abundance of the ascomycotic *Eurotiomycetes* spp. and particularly less of the zygomycotic *Mucor* spp. might actually protect against an unhealthy metabolic profile [12, 13]. By contrast, IBD patients host lower concentrations of *Saccharomyces cerevisiae* and more *Candida albicans* than healthy subjects do. In this disorder, an increased Basidiomycota-Ascomycota ratio is also observed, along with increased fungal diversity and richness [14, 15]. Interestingly, many IBD and obesity patients also produce anti-*S. cerevisiae* antibodies [1, 16, 17], although they host different abundance levels of these gut fungi. Taken together, these findings imply that the gut mycobiota could be aetiologically important in human diseases.

An understanding of the role of the gut mycobiota is emerging in relation to physiological as well as pathophysiological processes, but there is little knowledge of how the mycobiome is shaped from early life. High abundances of the genera *Penicillium*, *Aspergillus* and *Candida* (species-non-specific) were found in 10 Italian children who were each sampled once from 0 to 2 years of age [18]. Additionally, some common species have been studied. *C. albicans* and *Malassezia* spp. are partly transferred vertically from mothers to their offspring [19–21], supporting the theory that fungi colonise the neonatal gut through the birth canal. Culturing has also shown that the *Candida* spp. prevalence in neonates is 23%, and it more than doubles to 50% within 4 months [20]. The paradigm of the sterile intrauterine environment is now shifting, and several studies confirm the prenatal presence of commensal bacterial taxa in the placenta and amniotic fluid and the possible transmission of these bacteria to the foetus long before birth [22–26]. Corresponding knowledge on fungi is scarce. Early life microbiomes can also be affected by maternal exposure during pregnancy, ranging from high-fat diets to probiotics [27, 28]. Generally, we know that the mycobiome may be shaped by bacteria-fungus interactions [29, 30], as well as by the diet, by probiotics and by antibiotic administration (as shown in mice) [7, 31–33]. However, to our knowledge, the settling of early mycobiota with respect to the quantity, diversity and association with the maternal mycobiome has not been described before nor has the probiotic impact on the mycobiota been investigated.

In this prospective cohort, we describe the gut mycobiota in 298 pairs of healthy pregnant women and offspring from birth to 2 years of age. We report major shifts in the fungal abundance and diversity within these populations, and it supports the idea of a succession of mycobiotic hosting from mother to child and over the first 2 years of life.

## Methods

### Material

We selected 298 mother-offspring pairs with at least one pair of faecal samples from mothers and offspring who participated in the Probiotics in the Prevention of Allergy among Children in Trondheim study (ProPACT) (Table 1). The ProPACT study is a population-based, randomised, placebo-controlled and double-blinded trial on probiotics from Trondheim, Norway, and it has been described in detail elsewhere [34, 35]. Briefly, the pregnant women who attended the regular Norwegian Antenatal Care Programme were asked to participate by completing questionnaires on their health and risk factors and by collecting faecal samples from themselves and their offspring. The health questionnaire details, including antibiotic administration, were collected at 36 weeks of gestation and 6 weeks, 1 year and 2 years after birth. Although we do not have details on the antibiotic administration in the offspring before 10 days of life, no offspring that were delivered by caesarean section received antibiotics during labour, but one developed septicaemia afterwards and was treated accordingly. The probiotic milk administration was double-blinded and randomly provided to one half of the pregnant population from 36 weeks of gestation until 3 months after birth. The probiotic milk contained 5 × 10^10^ colony-forming units (CFUs) of *Lactobacillus rhamnosus* GG (LGG), 5 × 10^10^ CFUs of *Bifidobacterium animalis* subsp. *lactis* Bb-12 and 5 × 10^10^ CFUs of *Lactobacillus acidophilus* La-5 per day. The remaining half received placebos in the form of heat-treated fermented skimmed milk with no probiotic bacteria.

**Table 1:** Maternal and offspring characteristics

Maternal age at delivery (years (SD))	29.6 (± 3.9)
Caesarean sections	12.8%
Probiotic users	49.4%
Antibiotic therapy during pregnancy	7.2%
Male offspring	46.4%
Gestational age (weeks (SD))	40.4 (± 1.5)
Birth weight (kg (SD))	3.6 (± 0.4)
Birth length (cm (SD))	50.7 (± 3.3)
Breast-fed at 3 months	97.1%
Formula-fed at 3 months	6.5%
Proportion of children receiving antibiotic treatment within	
- 6 weeks	6.3%
- 1 year	17.2%
- 2 years	44.4%

A total of 1516 faecal samples were collected (Table 2). Maternal samples were collected at 35–38 gestational weeks of pregnancy and 3 months postpartum. Offspring faeces were obtained at 10 days, 3 months, 1 year and 2 years, and they were sampled from the diapers. Faecal samples were stored in a Cary-Blair transport medium, immediately frozen to − 18 °C at home, and collected and held in a frozen state until their permanent storage at − 80 °C before further analyses.

**Table 2:** DNA quantification and 18 rRNA gene ITS1 region sequencing of faecal samples

	Pregnant	Postpartum	10 days	3 months	1 year	2 years	Total
All faecal samples (count)	248	253	274	246	247	248	1516
Detected fungal ITS1	221 (89%)	220 (87%)	153 (54%)	148 (60%)	163 (66%)	189 (76%)	1094 (72%)
Sequenced samples	47 (19%)	27 (11%)	28 (10%)	4 (2%)	7 (3%)	12 (5%)	125 (8%)
Passed rarefaction and taxonomic classification	28 (11%)	25 (10%)	15 (6%)	4 (2%)	7 (3%)	11 (4%)	90 (6%)

#### Quantification

We used a protocol for bacterial DNA extraction that involved mechanical and chemical cell lysis. The stool samples were homogenised by bead beating with acid-washed glass beads (Sigma). We isolated the DNA with an LGC mag nucleic extraction maxi kit (LGC Genomics, Middlesex, UK) together with a KingFisher FLEX magnetic particle processor (ThermoScientific, Waltham, MA) according to the manufacturer’s recommendations, including a negative control as contamination control. Fungal internal transcribed spacer 1 (ITS1) amplicons were constructed using the primer pairs ITS1F (CTTGGTCATTTAGAGGAAGTAA) and ITS2 (GCTGCGTTCTTCATCGATGC), according to Tang et al. [36]. The fungal ITS quantities in 1516 samples were assessed with a LightCycler qPCR (Roche) of 50 cycles, using thermocycles comprising 95 °C in 15 min, then (95 °C in 30 s, 56 °C in 30 s, 72 °C for 45 s) × 50. For each qPCR plate, we included positive and negative controls (*S. cerevisiae* and sterile water, respectively). The qPCR cycle threshold (CT) value cut-off for fungal detection was set to either within the value of the negative control or to 45 cycles, because DNA quantification beyond 45 cycles can produce misleading results.

#### Sequencing of the 18S rRNA gene ITS1 region

Since many of the samples had low ITS DNA quantities, we chose to sequence the 18S ribosomal RNA (rRNA) gene ITS1 region of only those samples with sufficiently high ITS DNA quantities. We used a CT value of less than 35 cycles as the cut-off for sequencing. For the sequencing preparation, we measured the ITS DNA concentrations of the 125 selected ITS DNA samples (in addition to four positive and four negative controls) with FLx 800 cse (Cambrex), and they were normalised with a Biomek 3000 (Beckman Coulter) and prepared for amplicon sequencing using Illumina MiSeq v3 600-cycle chemistry, according to the producer’s instructions. Four positive and four negative controls were also included to the library. The library was quantified by using a Droplet Digital PCR (ddPCR, BioRad) and then diluted to a concentration recommended for sequencing. We then performed gene paired-read sequencing of the 18S rRNA gene ITS1 region on a MiSeq platform (Illumina). Resulting sequencing reads were first filtered out based on the quality score (minimum average *q*-score 25) and the barcode (no mismatches in the barcode were allowed). Remaining sequences were then pair-end joined and further filtered through UPARSE algorithm (max expected error (maxEE) value set to 0.25). The sequencing of the 18S rRNA gene ITS1 region produced a total of 3,722,830 reads. The median number of reads per sample was 16,355 reads and the mean was 27,991 reads, ranging from 3 to 119,463 reads per sample. We then used 6000 reads per sample as a cut-off for the rarefaction to ensure even representation of each sample in the dataset. The final dataset comprised 100 samples with more than 6000 sequences per sample and a total of 214 operational taxonomic units (OTUs). Ten of the samples were later discarded due to incorrect inclusion criteria, resulting in the inclusion of 90 samples in the analysis (Table 2).

We used the QIIME (Quantitative Insights into Microbial Ecology) pipeline for quality filtering and diversity estimation, whereas the UPARSE algorithm was used for OTU clustering [37]. In applying the rarefaction cut-off at 6000 reads per sample, we ensured minimal losses in the number of samples whilst maintaining the diversity (Additional file 1: Table S1 and Additional file 2: Figure S1).

The alpha diversity refers to the fungal diversity within each sample, and it was calculated by using Simpson’s reciprocal index, which describes how many OTUs prevail in each sample [38]. The beta diversity expresses the difference between the samples in terms of the number and abundance of OTUs within an age group, and it was calculated with the Bray-Curtis dissimilarity index.

Since there is no well-established quality annotation database designed for mycobiotic taxonomy assignment at present, and because fungi are often subject to misclassification, we used a conservative concordance system for the taxonomic annotation. We compared the OTU sequences with the four databases as follows: GenBank (NCBI, ≥ 97% identity and *E* value < 10^−50^); the Warcup Fungal ITS and UNITE Fungal ITS (User-friendly Nordic ITS Ectomycorrhiza Database with a bootstrapping threshold of 80%) through the Ribosomal Database Project (RDP) Classifier (https://rdp.cme.msu.edu/classifier/classifier.jsp); and the Targeted Host-Associated Fungi ITS Database (THF) [36], which was especially curated for the gut mycobiome (see Additional file 3 and Additional file 4). A concordance of at least three of these databases at the lowest taxonomic level or two databases with a justifiable was determined as sufficient qualification for the final assignment of each OTU. We followed the recent taxonomic reclassification for the fungi by manually curating the classification of OTU representative sequences with Index Fungorum as the reference [39].

#### Statistical methods

A standard curve was made to convert the qPCR CT values into fungal ITS copy concentrations in the faecal samples. The averages of three dilutions of the positive control for each qPCR plate of known fungal concentrations of *S. cerevisiae* were used for the calculation (see Additional file 5). The fungal ITS copy concentrations were logarithmically expressed to obtain a normal distribution. The fungal DNA data from the offspring samples were analysed using a linear mixed model for the fungal DNA concentration and a mixed logistic regression for the presence of fungal DNA. The models included a random intercept for mother-offspring pair, and age, maternal fungal DNA concentration/presence, the mode of delivery and maternal probiotics use were used as covariates. The effect of antibiotic use was studied in a separate model because we lacked information on the antibiotic use within 10 days of life. The interaction terms were investigated and included in the final analyses if significant. For independent data, multiple linear regression analyses were performed to test the associations between the fungal DNA abundance and clinical characteristics. The diversities and OTU abundances between the groups were analysed with non-parametric Mann-Whitney *U* tests and Kruskal-Wallis tests. By this, we disregard the potential within-subjects correlations; nevertheless, a non-parametric test for repeated measurements, i.e. Friedman’s test was not applicable due to the low numbers of observations in some groups. We defined the statistical significance as *p* < 0.05 and corrected the non-parametric analyses for multiple testing by controlling the false discovery rate through the Benjamini-Hochberg procedure. The statistical analyses were conducted using MATLAB 2016a (The MathWorks, Inc.), STATA 14 (StataCorp) and SPSS Statistics version 23.0 (IBM).

## Results

### Fungal DNA concentration and diversity

**Fig. 1:**
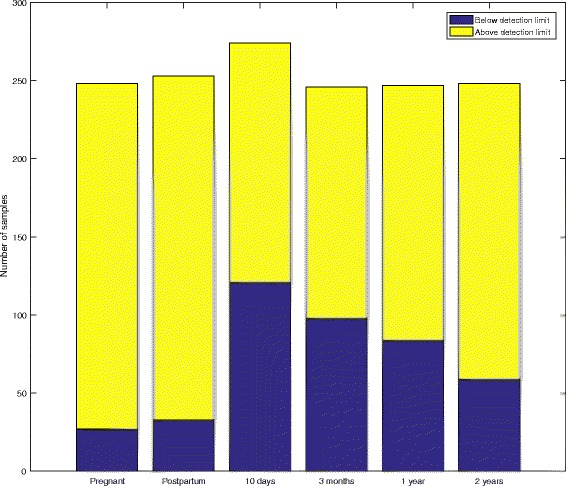
Detection of fungal ITS DNA. The counts of samples with detected and non-detected fungal ITS DNA for each age group. The detection limit was set to a higher fungal ITS concentration than the negative control or within a CT value of 45 cycles

**Fig. 2:**
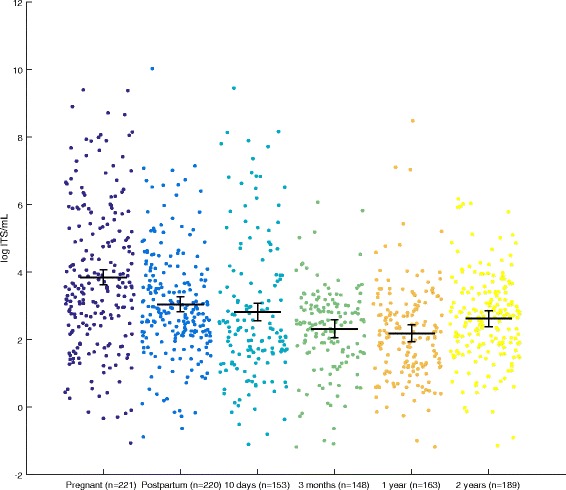
Fungal ITS DNA concentration in maternal and offspring faecal samples. A scatter plot of the fungal ITS DNA concentrations (log ITS copies per mL, mean and 95% CI). The concentration of the ITS copies quantifies the amounts of fungi in the samples

In total, 88% of the mothers and 56–76% of the offspring had detectable gut fungi (Fig. 1). The total fungal abundance was quantified by the amount of fungal ITS DNA copies in the sample*.* The samples from the pregnant women had the highest fungal DNA concentrations of all the groups (3.85 log(copies/mL) and 95% CI 3.62–4.07), which were significantly higher than those of the postpartum mothers (3.05 and 2.82–3.26, *p* < 0.001) (Fig. 2, Additional file 1: Table S2). Among the offspring samples, there was a tendency to uncover the highest fungal DNA concentration at 10 days (2.81 and 2.55–3.08), which then fell to the lowest levels at 1 year (2.19 and 1.94–2.45) before an increase at 2 years.

**Fig. 3:**
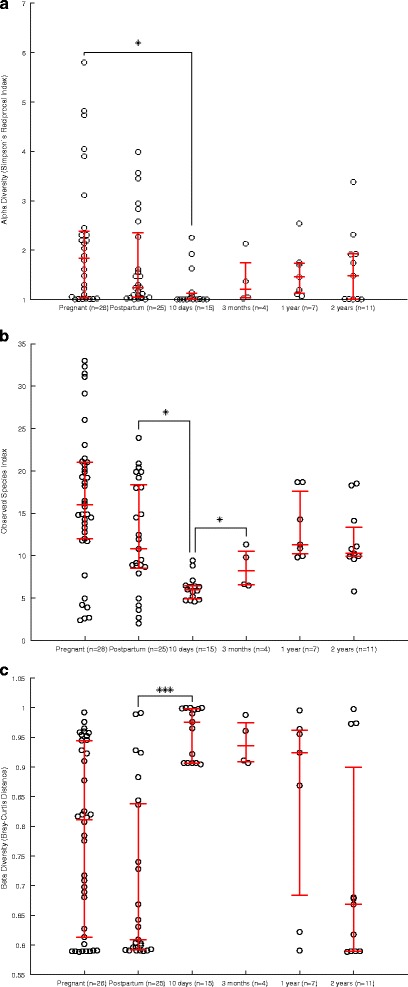
Alpha and beta diversity for the faecal samples. A scatter plot of the diversities; *red whiskers* designate the median and interquartile ranges. **a** Alpha diversity as Simpson’s reciprocal index. The Simpson reciprocal index describes how many OTUs prevail in each sample [36]. **b** The observed species index describes the sample richness, i.e. how many OTUs are detected in each sample. **c** The beta diversity as Bray-Curtis Distance describes the between-sample diversity from 0 to 1

Among all the groups, the alpha diversity was lowest in the 10-day samples (1.21, *p* < 0.05, Simpson’s reciprocal index) (Fig. 3a) and tended to be higher in the pregnant women (2.16, ns). This finding indicates that only 1–3 fungal species prevailed in mothers as well as offspring. The alpha diversity showed a consistent tendency to increase from birth to 2 years, but it did not reach significance due to the low number of samples in each group. A similar distribution was observed for the mean number of OTUs in each age group (Fig. 3b). In contrast to the alpha diversity, the beta diversity was highest at 10 days after birth (0.97, median Bray-Curtis dissimilarity index) and showed a spread between 0.6 and 1.0 in the other maternal and offspring samples (Fig. 3c). In summary, the mothers tended to have both higher alpha and beta diversity in pregnancy than postpartum. In the offspring, the alpha diversity seemed to increase steadily from birth, whereas the beta diversity was highest in 10-day-old offspring.

The PCoA plot at 6000 reads distinguished the 10-day–3-month-old offspring (breast-fed and/or formula-fed) from the other samples. Conversely, the samples from the 1–2-year-old offspring (fed a diet more similar to their mothers) converged towards the maternal pattern (Additional file 6: Figure S2).

### OTU taxonomic classification

In applying our stringent annotation method, 140 out of 245 OTUs were annotated at least up to fungal phylum, and 101 OTUs were classified by genus (Additional file 4).

**Fig. 4:**
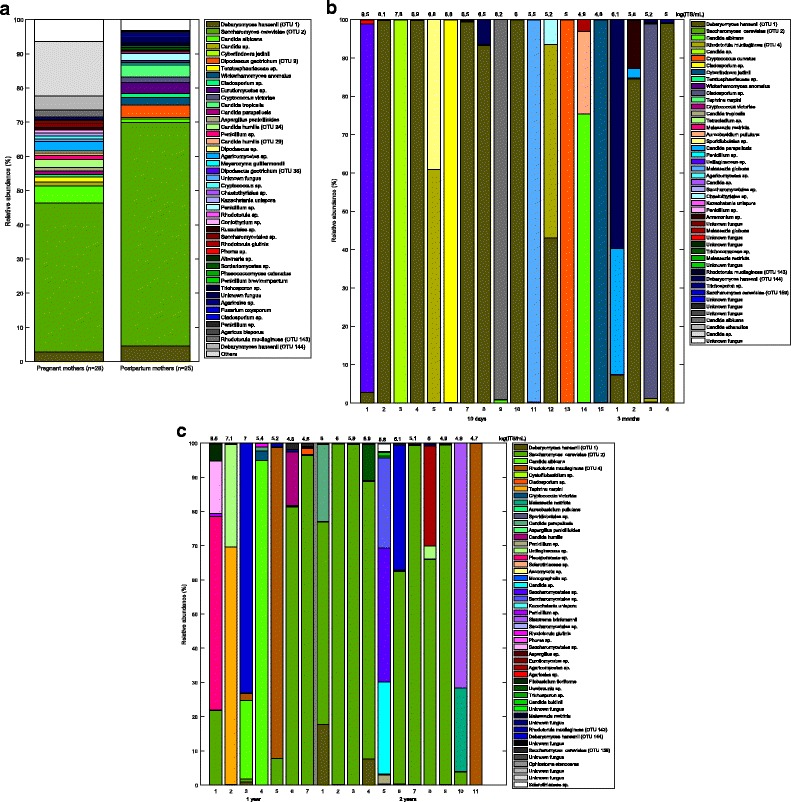
**a**–**c** OTU abundances for all groups. Bar charts of the relatively most abundant OTUs in **a** mothers, **b** offspring from 10 days to 3 months and **c** offspring for 1–2 years. Each *coloured box* represents an OTU. The individual fungal ITS DNA concentration is on *top* of each bar

Twelve OTUs differed significantly in their abundance between age groups, whilst they made up > 1% of the total relative abundance (Additional file 7: Figure S3). *S. cerevisiae* was most abundant in mothers and in offspring from 1 year of age onwards, whereas it was detected in very low quantities in offspring at 10 days and 3 months after birth. *Debaryomyces hansenii* exhibited its greatest abundance in offspring at 10 days and 3 months (Fig. 4a–c). The 10-day, 1-year and 2-year samples were richer in *Rhodotorula mucilaginosa*, whereas the 3-month samples showed a greater presence of *Candida parapsilosis* and a *Cladosporium* sp.

*Ascomycota* spp. comprised 86.4% of the fungi in all the age groups, with no significant difference between the age groups (*p* = 0.74). In total, 88.6% of the fungal community consisted of yeast species with no significant difference between the age groups (*p* = 0.60).

### Transfer of fungi from the mothers to the offspring

The odds of detecting fungal DNA in the offspring samples increased if the mothers also had detectable fungal DNA (odds ratio (OR) = 1.54 (95% CI 1.01–2.34, *p* = 0.04)) compared to mothers with no detectable fungi upon mixed logistic regression (Additional file 1: Table S3). Investigating the interactions, this effect was strongest 10 days after birth (OR = 3.7 (1.24–11.0), *p* = 0.019). In particular, we observed no effects of mode of delivery nor did we see any effects of maternal probiotic use or offspring antibiotic use. The intraclass coefficient (ICC) was < 0.01, which indicates that the repeated measurements were largely unrelated.

We found no significant associations between the offspring fungal DNA concentrations and the maternal fungal DNA concentrations, probiotics, mode of delivery or the offspring antibiotic use (Additional file 1: Table S4). The ICC was < 0.01, which shows that the repeated measurements were largely unrelated.

**Fig. 5:**
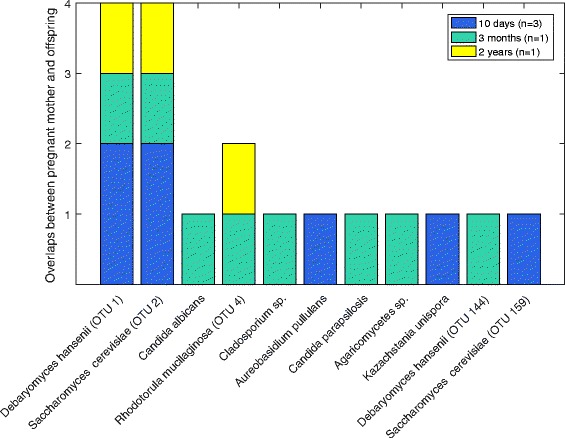
Overlapping OTUs between pregnant woman and their offspring. The counts of overlapping OTUs in five mother-offspring pairs from the sequencing of the 18S rRNA gene ITS1 regions

By sequencing the 18S rRNA gene ITS1 regions of merely high-quantity samples, 5 mother-offspring pairs remained. In these pairs, there were 11 overlapping species, with *D. hansenii* and *S. cerevisiae* being the most frequently overlapping ones (Fig. 5). These two species were also retained between pregnant and postpartum mother pairs (data not shown). Several other species were also present in the mother-offspring overlap, and these mostly belonged to the *Saccharomycetaceae* family, including *Candida* spp., in addition to *R. mucilaginosa*, *Malassezia* spp. and *Cladosporium* spp*.*

### Probiotics and fungal DNA concentrations in the mothers and the offspring

The pregnant mothers who were randomised to receive probiotics had significantly increased fungal DNA concentrations compared to the controls (*p* < 0.01, Additional file 1: Table S5). Adjusting for a history of antibiotic treatment did not change the effect estimator. One *S. cerevisiae* strain (OTU 159) tended to be underrepresented in the probiotic-receiving pregnant women (*p* = 0.07); however, the distributions of the other *S. cerevisiae* strain (OTU 2) remained the same (Additional file 8: Figure S4).

## Discussion

In this study, we showed that the gut fungi are detectable in most mothers and the majority of their offspring already at the age of 10 days. *S. cerevisiae* was the most abundant fungal species in mothers and 1- to 2-year-old offspring, whereas *D. hansenii* prevailed during the first months of life. Furthermore, there was an increased risk of fungal DNA presence in offspring if the mother had detectable fungi. We also found that the maternal fungal DNA concentrations increased when the pregnant mothers drank probiotics.

Almost 90% of the mothers and 60–80% of the offspring had detectable gut fungi, which highlights fungi as an inherent part of the gut microbiota. These proportions cohere with the findings of an Italian cross-sectional fungal cultivation study on children and adults [18].

Many of the fungal species that we detected in the offspring have previously been detected in the adult gut (see Additional file 5). Strikingly, *D. hansenii*, an ubiquitous *Saccharomycetaceae* species that is often used in the food industry as a cheese yeast [40], dominates the offspring gut mycobiota during the breast-feeding period. *D. hansenii* can stem from breast milk because this fluid was the only dietary component at 3 months, and similar yeasts (*C. albicans*) have been found in breast milk before [41]. *D. hansenii* has also been found on the facial skin of children [42]. *D. hansenii* has previously been cultured from human faeces and has been shown to grow well in milky environments like cheese and possibly breast milk, since some strains are known to grow at 37 °C [20, 40]. All these findings suggest that *D. hansenii* could be an autochthonous species of the early mycobiota.

We found *S. cerevisiae* in the newborn offspring, which has not been shown before. Intriguingly, *S. cerevisiae* first surges and then becomes a dominant species at 1 year of age, after the introduction of food containing *S. cerevisiae* (e.g. bread) into the diet. This finding suggests both a birth-related and a dietary means of colonisation. *S. cerevisiae* is present in most diets and has been found and cultured from faecal samples. However, it is also capable of causing opportunistic infections [20, 43], implying that it is an autochthonous species. We observed that *S. cerevisiae* was substantially more abundant in offspring and adult gut mycobiota than previously described [1, 9], and there was a relatively low occurrence of *Candida* spp.; this variation may be due to different regional diets, host genetics or fungal detection methods. Some studies have shown that by using culture-dependent techniques, one would obtain relatively more *Candida* spp. than when using culture-independent techniques [2, 18], which could partly explain the rather limited amounts of *Candida* spp. in our study.

Some of the identified fungi should be regarded as transients that do not colonise the gut. The OTUs *Agaricomycetes*/*Agaricales* sp. are likely from edible and non-colonising fungi, e.g. the button mushroom *Agaricus bisporus*, and similarly, *Ustilaginaceae* spp. are well-known plant pathogens. These fungi are generally of lower quantities and are often known not to live in anaerobic and body temperature environments; they are thus most likely transients from food. Not all OTUs were annotated to the species level because the fungal databases still are under development. However, our strict classification improved the chance of an accurate classification.

We found that maternal fungal hosting makes the offspring more inclined to host fungi. This effect was strongest at 10 days after birth. The increased chance of fungal hosting suggests that these mother-offspring pairs share physiological fungal hosting abilities. Because fungi are ubiquitous in the environment, they may originate from the mother during birth, the mother’s breast milk, parental skin or anywhere else in the hospital or home environment with which the offspring come in contact. However, we did not observe any OTU abundance difference regarding vaginal or caesarean delivery. Thus, we find indications for the transfer of fungal hosting between mothers and offspring that appear to be independent of the mode of delivery.

The overlapping OTUs in the mother and offspring guts were mostly *Saccharomycetaceae* spp. This fungal family seems to have adapted well to the human gut environment and may be the species that are fittest to survive the transfer into the newborn fungal host environment. In addition to the gut mycobiota, *Saccharomycetaceae* spp. are also the most abundant species in healthy human mouth mycobiota [44].

The fungal abundance varied by age, which supports the idea that physiological fungal succession occurs in the early gut mycobiota. We have shown that the gut mycobiota is establishing already at 10 days after birth, albeit at a lower abundance and diversity than what is detected in their mothers’ guts. At 10 days, the gut fungi have just started to fight for their positions in a seemingly first-come first-serve model; the reduction in fungal abundance is probably determined by feeding, gut immunity and their interactions. The decrease at 3 months may be due to a previously described temporarily high abundances of *Bifidobacterium* spp. and *Lactobacillus* spp. that exhibit fungal antagonism [30, 45]. Upon approaching 2 years of age, the gut mycobiota consists of fungi specific for the adult mycobiota, as observed in our study.

Interestingly, pregnant mothers that received probiotics showed a higher abundance of gut fungi. This finding could indicate that the probiotic bacteria used in our study promote the symbiotic growth of gut fungi, like other lactic acid bacteria that are known to grow mutually with yeasts [46].

Due to a strict ITS quantity cut-off, a smaller proportion of the samples was sequenced. It cannot be excluded that the lower-abundance samples have different compositions. Nevertheless, in this unselected study population of mothers and offspring, it is reasonable to surmise that this selection could reflect the gut mycobiota of a healthy gut. The study design gave us limited control of the faecal sampling, but all the mothers were well informed about how to collect and quickly freeze the samples to avoid contamination and improve preservation. In the future, a lysis protocol optimised for fungal DNA extraction would be preferable, but this approach would require the fungal extraction analysis of a representative selection of human-associated fungi that is not yet available.

## Conclusion

Our findings provide the first insight into the gut mycobiota that is established in offspring and into the transfer of fungal hosting from mother to child. This study covers a large, unselected population cohort of mothers and offspring, and it broadens the field of gut mycobiota as a new research area. The ways in which the early mycobiota can affect a child’s normal physiology with respect to growth, immunity and metabolism remain to be elucidated.

## Additional files

Additional file 1: Table S1. Rarefaction table of sequenced samples. **Table S2.** ITS DNA concentration for all age groups. **Table S3.** Detectable fungal DNA in offspring according to maternal characteristics and offspring age. **Table S4.** Fungal DNA concentration in offspring according to maternal presence and offspring age. **Table S5.** Association between fungal DNA concentration in pregnant mothers and maternal use of antibiotics and probiotics. (XLSX 16 kb)

Additional file 2: Figure S1. Number of samples vs. rarefaction cut-off. To compare the samples, a rarefaction is performed to obtain the same number of sequences in each sample. By increasing the rarefaction cut-off, the number of observed species increases with the sacrifice in the number of included samples. Using 6000 sequences as the rarefaction cut-off is a reasonable trade-off. (PDF 6 kb)

Additional file 3: Concordance of fungal databases and final assignment. (XLSX 101 kb)

Additional file 4: Table of fungal species. (XLSX 18 kb)

Additional file 5: Concentration and standard curve calculations. (DOCX 32 kb)

Additional file 6: Figure S2. Principal coordinates analysis (PCoA). A PCoA plot at 6000 reads per sample. (PDF 8 kb)

Additional file 7: Figure S3. Significantly different OTUs between groups. A significantly different abundance of OTUs between groups in terms of relative abundance, as tested by Kruskal-Wallis test. Each bar represents an OTU, for which the relative abundances of all the groups are added. Only species > 1% of the relative abundance in at least one age group are included in the analysis. (PDF 5 kb)

Additional file 8: Figure S4. OTU abundances for probiotics in pregnant mothers. The OTU abundances in pregnant mothers with and without probiotics. Each coloured box represents an OTU. (PDF 5 kb)

## Abbreviations

CFU: Colony-forming unit
CI: Confidence intervals
CT: Cycle threshold
ddPCR: Droplet Digital Polymerase Chain Reaction
IBD: Inflammatory bowel disease
ICC: Intraclass coefficient
ITS: Internal transcribed spacer
OR: Odds ratio
OTU: Operational taxonomic unit
ProPACT: Probiotics in the Prevention of Allergy among Children in Trondheim Study
PRR: Pathogen-recognition receptor
QIIME: Quantitative Insights into Microbial Ecology
qPCR: (Real-time) quantitative polymerase chain reaction
SD: Standard deviation
Sp. and spp.: Species (singular) and species (plural)
