# Correction to: Early gut mycobiota and mother-offspring transfer

**DOI:** 10.1186/s40168-021-01094-x

**Published:** 2021-05-21

**Authors:** Kasper Schei, Ekaterina Avershina, Torbjørn Øien, Knut Rudi, Turid Follestad, Saideh Salamati, Rønnaug Astri Ødegård

**Affiliations:** 1 Department of Laboratory Medicine, Children’s and Women’s Health, Faculty of Medicine and Health Sciences, NTNU – Norwegian University of Science and Technology, Postboks 8905, 7491 Trondheim, Norway; 2 Department of Chemistry, Biotechnology and Food Science, NMBU – Norway University of Life Sciences, Ås, Norway; 3 Department of Public Health and Nursing, Faculty of Medicine and Health Science, NTNU – Norwegian University of Science and Technology, Trondheim, Norway; 4 ObeCe – Regional Centre for Obesity Research and Innovation, St. Olav’s University Hospital, Trondheim, Norway

**Correction to: Microbiome 5, 107 (2017)**

**https://doi.org/10.1186/s40168-017-0319-x**

Following publication of the original article [1], the authors report an error in a sub-analysis of the effect of probiotic milk on fungal abundance in *maternal faecal samples from pregnancy weeks 36–38*. Most samples were obtained before the probiotic intervention, not after as we originally reported and a correct approach would be to regard maternal pregnancy faecal samples as baseline data.

Accordingly the following text should be deleted: in the Abstract result paragraph (page1); “Probiotic consumption increased the gut mycobiota abundance in pregnant mothers (*p* = 0.01)”, in the Result section (page 6); “Probiotics and fungal DNA concentrations in the mothers and the offspring. The pregnant mothers who were randomised to receive probiotics had significantly increased fungal DNA concentrations compared to the controls (*p* < 0.01, Additional file 1: Table S5). Adjusting for a history of antibiotic treatment did not change the effect estimator. One *S. cerevisiae* strain (OTU 159) tended to be underrepresented in the probiotic-receiving pregnant women (*p* = 0.07); however, the distributions of the other *S. cerevisiae* strain (OTU 2) remained the same (Additional file 8: Figure S4).”, and in the Discussion section (page10); “Interestingly, pregnant mothers that received probiotics showed a higher abundance of gut fungi. This finding could indicate that the probiotic bacteria used in our study promote the symbiotic growth of gut fungi, like other lactic acid bacteria that are known to grow mutually with yeasts.” In Additional files Figure S4 should be deleted. In Table S5 the title currently reads “Association between fungal DNA concentration in pregnant mothers and maternal use of antibiotics and probiotics”. It should read “Association between fungal DNA concentration in pregnant mothers and maternal use of antibiotics”. The first row in this table; “Maternal probiotics during late pregnancy” should be deleted.

## References

[1] Schei K, Avershina E, Øien T, Rudi K, Follestad T, Salamati S (2017). Early gut mycobiota and mother-offspring transfer. Microbiome..

