# Supplementary figures and images for: Early gut mycobiota and mother-offspring transfer
Source: Microbiome. 2017 Aug 24;5:107. doi: 10.1186/s40168-017-0319-x (PMC5571498; doi:10.1186/s40168-017-0319-x)

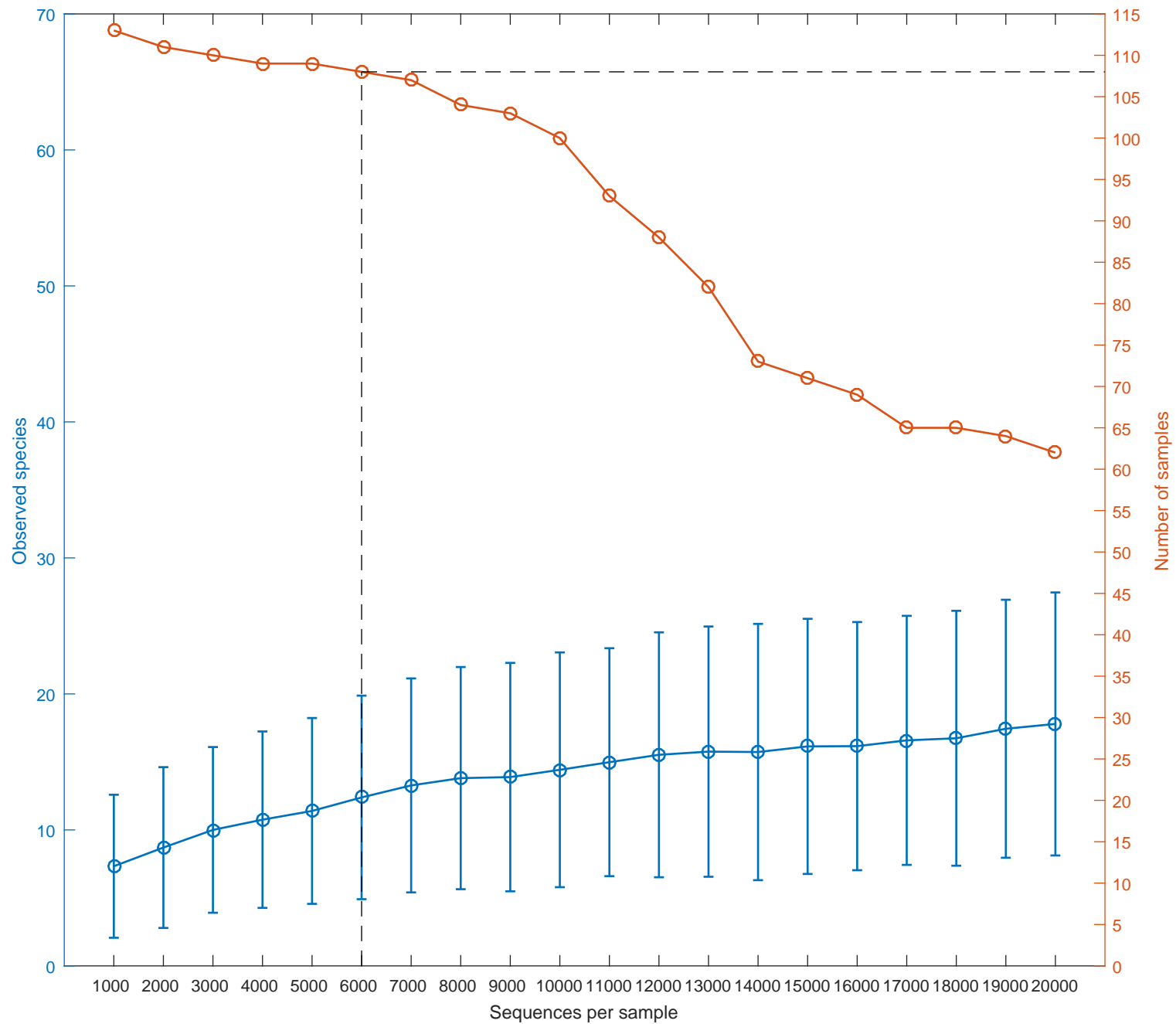

Supplement: Supplementary file 2 — Number of samples vs. rarefaction cut-off. To compare the samples, a rarefaction is performed to obtain the same number of sequences in each sample. By increasing the rarefaction cut-off, the number of observed species increases with the sacrifice in the number of included samples. Using 6000 sequences as the rarefaction cut-off is a reasonable trade-off. (PDF 6 kb) [file 40168_2017_319_MOESM2_ESM.pdf]

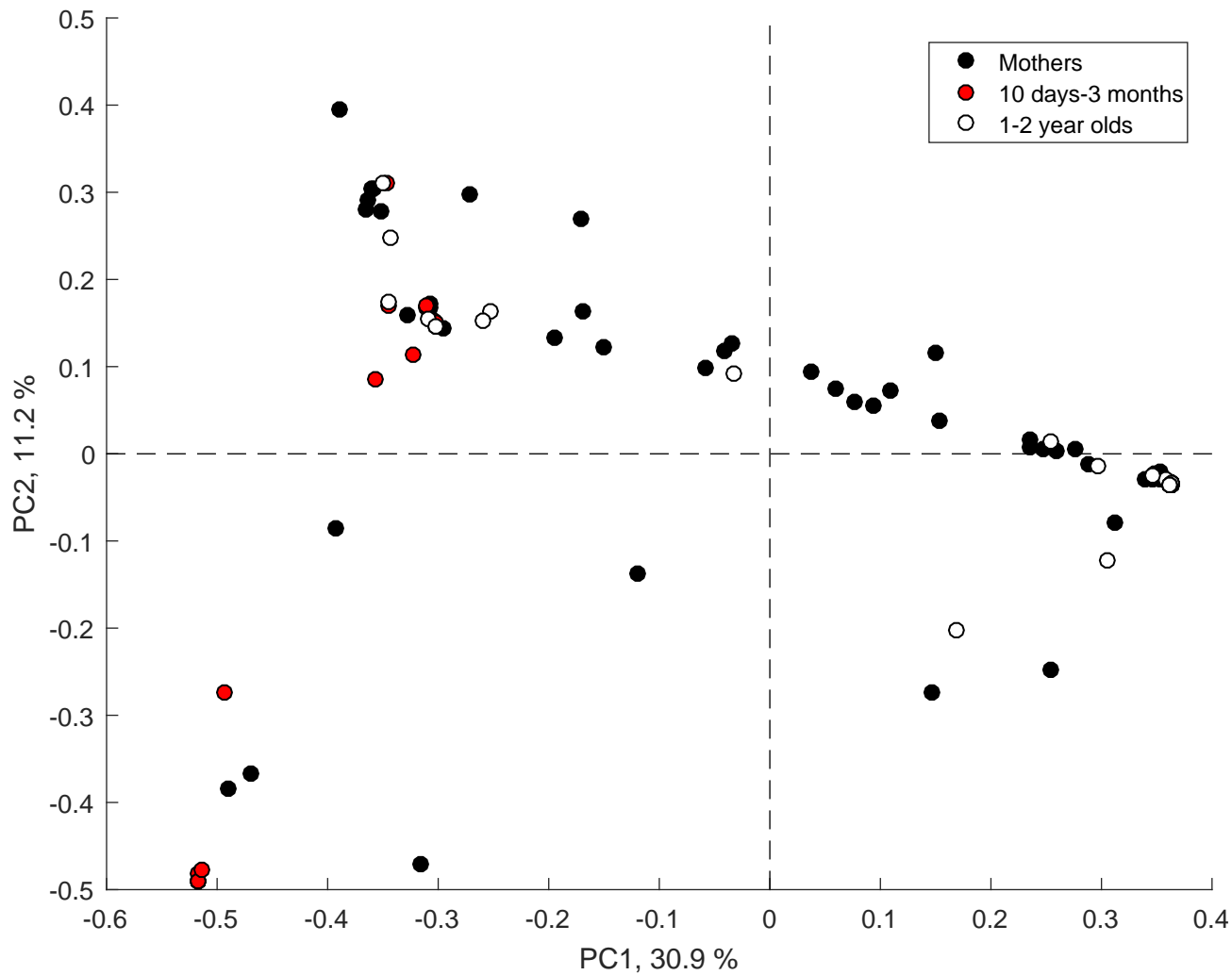

Supplement: Supplementary file 6 — Principal coordinates analysis (PCoA). A PCoA plot at 6000 reads per sample. (PDF 8 kb) [file 40168_2017_319_MOESM6_ESM.pdf]

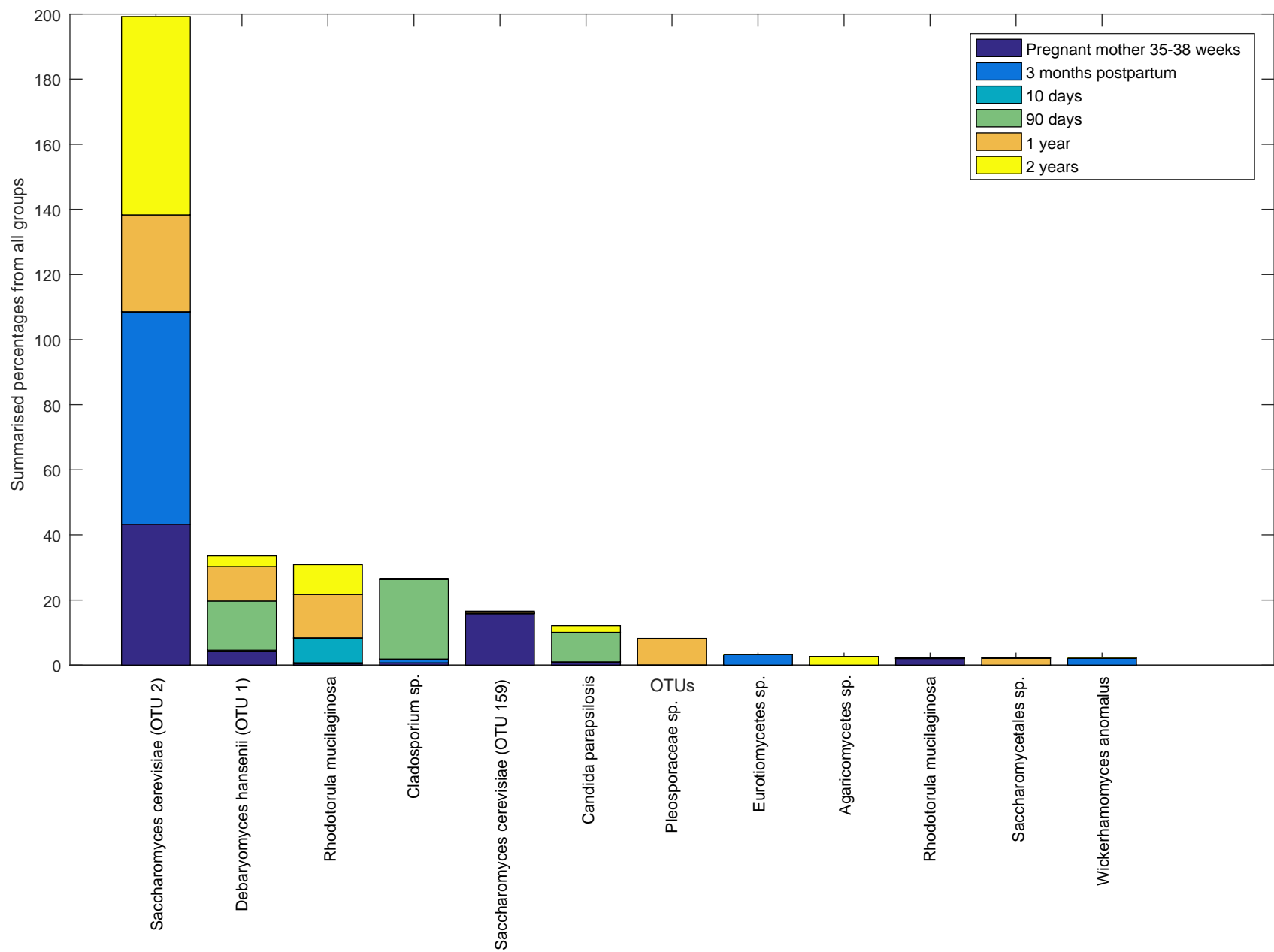

Supplement: Supplementary file 7 — Significantly different OTUs between groups. A significantly different abundance of OTUs between groups in terms of relative abundance, as tested by Kruskal-Wallis test. Each bar represents an OTU, for which the relative abundances of all the groups are added. Only species > 1% of the relative abundance in at least one age group are included in the analysis. (PDF 5 kb) [file 40168_2017_319_MOESM7_ESM.pdf]

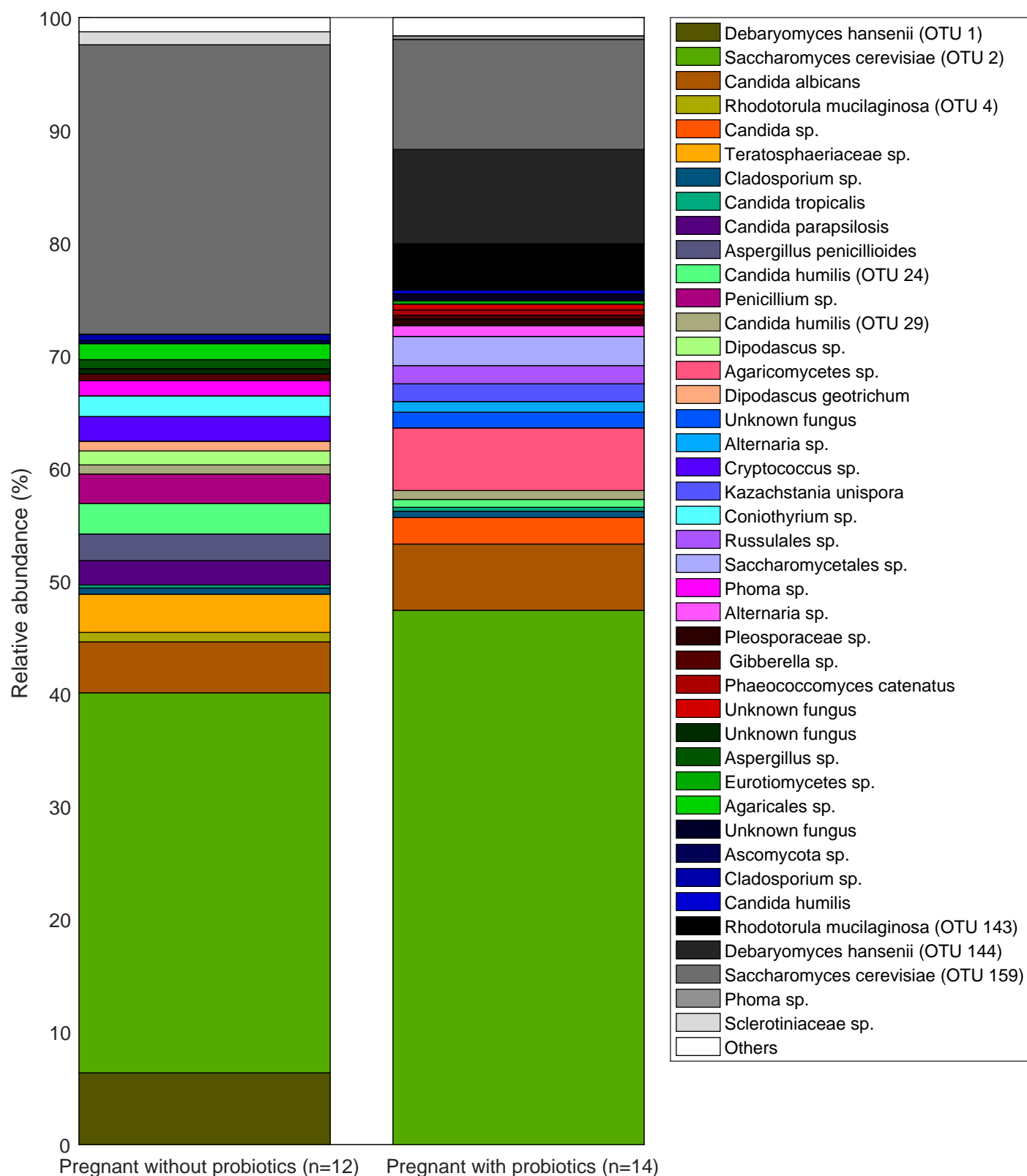

Supplement: Supplementary file 8 — OTU abundances for probiotics in pregnant mothers. The OTU abundances in pregnant mothers with and without probiotics. Each coloured box represents an OTU. (PDF 5 kb) [file 40168_2017_319_MOESM8_ESM.pdf]
