# Supplementary material for: Early gut mycobiota and mother-offspring transfer
Source: Microbiome. 2017 Aug 24;5:107. doi: 10.1186/s40168-017-0319-x (PMC5571498; doi:10.1186/s40168-017-0319-x)
Supplement: Supplementary file 5 — Concentration and standard curve calculations. (DOCX 32 kb) [file 40168_2017_319_MOESM5_ESM.docx]

# Concentration and Standard Curve Calculations

To convert the CT values into ITS DNA concentration, we created a positive control of known fungal concentration that was integrated in the analyses. The positive control was made by carefully dissolving a commercially produced fresh baking yeast block consisting of a *S. cerevisiae* strain into sterile water, and we then extracted the DNA in the same manner as the DNA from the faecal samples were extracted. Then, we quantified the DNA concentration of the positive control with Qubit (Thermo Fisher Scientific). We next calculated the concentration of ITS copies in the positive control, as shown below. Then, we made three dilutions of the positive control, which we included in all 17 PCR plates. We quantified the CT values of these dilutions with the qPCR in the same way as the faecal sample CT values were quantified (see the method section), and thereafter used the average of the CT values to make a linear standard curve. Since the positive controls were diluted by tenths, we used the logarithmic function of the ITS concentration to obtain a linear standard curve. This standard curve made it possible to convert CT values to ITS concentration in the other samples.

**DNA concentration in positive control**Concentration in positive control of *Saccharomyces cerevisiae* with Qubit: 0.102 µg/mL.

**ITS copy number in 1 mL of *S. cerevisiae* (positive control)**Total ITS length: 500-600 bp.
ITS1F-ITS2: 314 bp (http://www.ncbi.nlm.nih.gov/pmc/articles/PMC4059633/)
*Saccharomyces cerevisiae* total genome: 12 157 105 bp (haploid) (<http://www.yeastgenome.org/strain/S288C/overview>)

$$Mass of S. cerevisiae genome=$$

$$m=n\times1.096\times\frac{{10}^{-21}g}{bp}$$

$$m=12157105 bp\times1.096\times\frac{{10}^{-21}g}{bp}$$

$$m=1.332418708\times{10}^{-14}g$$

$$m=0.013324 pg$$

There is only one copy number of ITS1 in a haploid genome. 0.0133 pg of fungal DNA thus contains one copy of ITS1.

Concentration in positive control: 0.102 µg/mL = 102 ng/mL = 102 000 pg/mL.

$$Mass of S. cerevisiae genome containing one copy=$$

$$m_{one}= 0.013324\frac{pg}{copy}$$

$$ITS copy concentration in positive control=$$

$$C_{ITS}=\frac{102000\frac{pg DNA}{mL}}{0.013324\frac{pg DNA}{copy}}=7655251.265\frac{ITS copies}{mL}$$

$$C_{ITS}=7 655 251\frac{ITS copies}{mL}$$

$${10}^{-3}dilution: 7655 \frac{ITS copies}{mL}$$

$${10}^{-4}dilution: 765.5 \frac{ITS copies}{mL}$$

$${10}^{-5}dilution: 76.55 \frac{ITS copies}{mL}$$

**ITS standard curve**Mean CT values for positive controls in all 17 plates were calculated. From the above calculated ITS concentrations in the three dilutions of the positive control, we constructed a normal curve based on ITS concentration (logarithmically expressed for normality) and CT values.

| Dilutions | ITS/mL (x) | log(x) | Mean CT values (y) |
| --- | --- | --- | --- |
| 10^-3^ | 7655 | 3.883945195 | 28.13 |
| 10^-4^ | 765.5 | 2.883945195 | 31.48 |
| 10^-5^ | 76.55 | 1.883945195 | 35.69 |

Above is the linear regression line calculated from 3 different dilutions of the positive control on all 17 PCR plates. Based on the regression line, the following equation was used to calculate the ITS copy number:

$$\log\left( \frac{ITS}{ml} \right)=\frac{42.664-CT value}{3.7787}$$
